# Food insecurity in females with phenylketonuria

**DOI:** 10.1002/jmd2.12115

**Published:** 2020-03-20

**Authors:** Kathryn E. Coakley, Suzanne Porter‐Bolton, Mary L. Salvatore, Rosalynn B. Blair, Rani H. Singh

**Affiliations:** ^1^ Nutrition and Dietetics Program; Department of Individual, Family, and Community Education University of New Mexico Albuquerque New Mexico USA; ^2^ Chief Turnaround Office Georgia Department of Education Atlanta Georgia USA; ^3^ Metabolic Genetics and Nutrition Program, Department of Human Genetics, Division of Medical Genetics Emory University School of Medicine Atlanta Georgia USA

**Keywords:** dietary intake, food insecurity, medical food, nutrition, phenylalanine, phenylketonuria

## Abstract

Phenylketonuria (PKU) is a genetic disorder characterized by insufficient metabolism of phenylalanine. Depending on severity, patients follow a low‐phenylalanine diet and may consume medical food (MF) and low‐protein modified foods; dietary and medical treatment can be expensive. This study assessed prevalence of food insecurity (FI), the lack of resources to access enough nutritious food to have an active, healthy life, in females with PKU and examined associations with diet and metabolic control. Participants were recruited from a research‐based camp in 2018. Adult and adolescent modules of the USDA Household Food Security survey were utilized to categorize participants as food secure [high food security (FS) or marginal FS] or food insecure (low FS or very low FS); results were compared to the general U.S. population. Dietary intake via three‐day food records and plasma amino acids were also assessed. Thirty females 11‐58 years of age (mean = 21.4 years) participated. Twelve (40%), including seven adolescents (44%) and five adults (36%), were FI compared to the U.S. prevalence of 11.1%. MF protein intake was significantly lower in those with very low FS compared to high FS and low FS (*P* = .04). Age and intact protein intake were significantly higher in those with very low FS compared to high FS (*P* < .05). Our study suggests adolescent and adult females with PKU have a higher prevalence of FI than the general U.S. population. Those with very low FS were older, consumed more dietary phenylalanine and intact protein, and less MF protein. Clinicians should consider screening for FI in patients with PKU.

**SYNOPSIS**

Food insecurity is more prevalent in adolescent and adult females with phenylketonuria (PKU) than the general U.S. population.

## 1 INTRODUCTION

Phenylketonuria (PKU), also known as phenylalanine hydroxylase deficiency (OMIM #261600), is a genetic disorder characterized by insufficient metabolism of the amino acid, phenylalanine. Patients with PKU follow a phenylalanine‐restricted diet, with the amount of intact protein from food prescribed based on the severity of the driver mutation in the phenylalanine hydroxylase gene.^1^ The goal of dietary treatment is to maintain blood phenylalanine concentrations within a therapeutic range (120‐360 μmol/L) to prevent severe physical, cognitive, and behavioral complications.^1^ Medical food is prescribed to supply a phenylalanine‐free protein source and patients also may purchase low‐protein modified foods (LPMF) as an alternate low‐phenylalanine energy source. Medical food is very expensive, costing up to 23 times more than infant formula.^2^ Insurance coverage for medical food and LPMF varies by state and families of patients may have to cover a portion of expenses.^3^

The annual cost of PKU treatment, including medical food and LPMF, varies by age but is estimated to range from $19 057 to $54 147.^4^ A recent study of families with children with any inherited metabolic disorder (IMD) found 21% of parents spent more than $100 per month for medical food and 48% spent more than $100 per month for LPMF.^2^ Only 40% of families reported insurance or other assistance in covering the costs of LPMF with 60% paying out of pocket.^2^ Advances in medical and pharmaceutical treatment of PKU have been life altering but also come with significant costs, though most medications are covered by insurance.

Food insecurity is a concern in households with significant medical expense related to chronic disease.^5^ The U.S. Department of Agriculture (USDA) defines food security for a household as access by all members at all times to enough food for an active, healthy life.^6^ Conversely, food insecurity is the limited or uncertain availability of nutritionally adequate and safe foods or limited or uncertain ability to acquire acceptable foods in socially acceptable ways.^6^ There are four categories of food security ranging from high food security, where a household has no problems or anxiety about consistently accessing adequate food, to very low food security, where eating patterns and food intake may be altered because of lack of money or resources for food.^6^

Food insecurity impacts physical and mental health in children and adults. In children, food insecurity is related to significantly worse general health, acute and chronic health problems, and healthcare access obstacles.^7^ In adults, food insecurity is associated with cardiovascular risk factors, obesity and comorbidities including diabetes and asthma, depression, and other health issues.^8^, ^9^, ^10^ Adults with PKU also have a higher risk for comorbid conditions including obesity, abnormal lipid profiles, and anxiety and depression.^11^, ^12^, ^13^ The combination of a chronic metabolic disorder like PKU and food insecurity could exacerbate health issues.

Food insecurity in individuals with PKU has not been reported. This study assessed the prevalence of food insecurity in females with PKU and examined associations between food security status and dietary intake and metabolic control.

## 2 SUBJECTS AND METHODS

### 2.1 Subjects

Females 11 years of age and older attending a research‐based camp for girls and women with PKU in 2018 were eligible for this study. Participants volunteer to attend camp and females 11 years of age and older are eligible to attend. Exclusion criteria included those not participating in camp research and those with a diagnosis of maple syrup urine disease (MSUD) who also attended camp. The study was approved by the Emory University Institutional Review Board (IRB) and data were collected in compliance with Health Insurance Portability and Accountability (HIPAA) guidelines. All participants provided informed consent prior to participating.

### 2.2 Study design

This was a cross‐sectional pilot study. Anthropometric measurements, dietary intake, and biochemical data were collected on the first day of camp at two research sites depending on participant age. All participants were fasting for at least two and a half hours before study visits. A trained phlebotomist drew venous blood for all blood samples and a research laboratory technician performed preliminary processing and aliquoting of the samples.

### 2.3 Food insecurity

The U.S. Department of Agriculture (USDA) U.S. Household Food Security Survey Module was utilized to assess food security in participants 18 years of age and older.^14^ This module includes 10 questions for all households and an additional eight questions for households with children. Questions assess food insecurity over the past 12 months. The USDA's Food Security Survey Module for Youth Ages 12 and Older was utilized to assess food security in participants under 18 years of age. This module includes nine questions and is validated in adolescent population.^15^ The USDA recommends utilizing a 30‐day reference period for the adolescent module. Both modules were scored per USDA guidelines to classify participants as high food security (HFS), marginal food security (MFS), low food security (LFS), or very low food security (VLFS). The four categories of food security can then be collapsed into food secure (HFS and MFS) or food insecure (LFS and VLFS) per USDA definitions. Both food security survey instruments are included in the Appendix1.

### 2.4 Dietary intake

Participants were instructed to record all food, beverages, medical food, and supplements consumed in the three days preceding camp. Three‐day diet records were submitted by all participants on the first day of camp and reviewed individually with a metabolic Registered Dietitian Nutritionist (RDN) to ensure completeness and accuracy. If participants did not bring a completed three‐day food record to camp, the RDN performed a 24‐hour recall. All food records and recalls were analyzed via Metabolic Pro. The three days of dietary intake were averaged to calculate mean daily intake of calories, total protein, protein from food (intact protein), and protein from medical food.

### 2.5 Other variables

Plasma amino acids were analyzed at Emory Genetics Laboratory Eurofins via ion exchange chromatography. Subjects also completed surveys on demographics, medications, medical food prescription (grams protein/day) and dietary phenylalanine prescription (mg phenylalanine/day). Height and weight were measured on a calibrated stadiometer and scale respectively to calculate body mass index (BMI). For adults, standard BMI definitions were utilized to classify participants as underweight, normal weight, overweight. or obese. For adolescents 11‐20 years of age, BMI‐for‐age percentiles were utilized to classify participants as underweight, normal weight, overweight, or obese, per Center for Disease Control and Prevention (CDC) definitions.

### 2.6 Statistical analysis

All statistics were performed using Statistical Analysis Software (SAS) version 9.4.^16^ Since the study was a cross‐sectional pilot study, a power calculation was not performed. Prevalence of the four categories of food security and the prevalence of food insecurity (LFS and VLFS) in the total sample, in adolescents, in adults, and in households with children were calculated and compared to prevalence in the United States. Differences in variables including age, BMI, blood phenylalanine and tyrosine, dietary intake, and phenylalanine prescription were assessed between the four categories (HFS, MFS, LFS, VLFS) via linear regression, controlling for age and medication use.

## 3 RESULTS

Thirty females 11‐58 years of age (mean = 21.4 ± 11.7 years) participated. Sixteen (53%) were under 18 years of age. Fourteen (47%) were 18 years of age or older with five of the 14 adults (36%) reporting children in their household. Mean BMI was 27.0 ± 8.7 kg/m^2^ and 60% were overweight or obese (BMI ≥ 25 for adults 20 years or older; BMI at the CDC's 85th%ile BMI‐for‐age or above for adolescents). Mean blood phenylalanine concentration was 601 ± 401 μmol/L. On average, participants reported a dietary phenylalanine prescription of 787 ± 684 mg per day. Half of the participants reported taking blood phenylalanine‐lowering medications including 14 (47%) taking sapropterin and one (3%) taking pegvaliase. Demographics are summarized in Table 1.

**Table 1:** Characteristics of female study participants (n = 30)

	Mean (SD)	n	Percent
Age (years)	21.4 (11.7)		
11‐17		16	53.3%
≥18		14	46.7%
Body mass index (BMI)	27.0 (8.7)		
Underweight (<18.5 or <5th%ile)		0	0%
Normal (18.5‐24.9 or 5‐85th%ile)		12	40.0%
Overweight (25‐29.9 or 85‐95th%ile)		8	26.7%
Obese (≥30 or >95th%ile)		10	33.3%
Plasma phenylalanine (μmol/L)	601 (401)		
Below treatment range (<120)		2	6.7%
Normal (120–360)		9	30.0%
Above treatment range (>360)		19	63.3%
Medications			
None		15	50.0%
Sapropterin		14	46.7%
Pegvaliase		1	3.3%
Dietary phenylalanine prescription (mg/day)	787 (684)		
Dietary Intake			
Total calories (kcals/day)	1548 (418)		
Total protein (g/day)	57.2 (16.3)		
Intact protein from food (g/day)	19.7 (17.4)		
Protein from medical food (g/day)	37.5 (21.0)		
Phenylalanine (mg/day)	894 (785)		

In the total sample, 12 (40%) were food insecure compared to the U.S. prevalence of 11.1% in 2018.^17^ Of the 12 who were food insecure, three (10% of the sample) were considered very low food security, much higher than the U.S. prevalence of 4.3%.^17^ Forty‐four percent of adolescents (n = 7) were food insecure and 36% of adults (n = 5) were food insecure. Sixty‐six percent (n = 3) of households with children were food insecure compared to the U.S. prevalence of 13.9%.^17^ Prevalence of food security is reported in Table 2.

**Table 2:** Food security status of female study participants (n = 30)

	Adolescents (n = 16) 11–17 years of age		Adults (n = 14) ≥18 years of age		Full sample (n = 30)	
	n	%	n	%	n	%
Food secure	9	56.3%	9	64.3%	18	60.0%
High food security (HFS)	8	50.0%	7	50.0%	15	50.0%
Marginal food security (MFS)	1	6.3%	2	14.3%	3	10.0%
Food insecure	7	43.8%	5	35.7%	12	40.0%
Low food security (LFS)	6	37.5%	3	21.4%	9	30.0%
Very low food security (VLFS)	1	6.3%	2	14.3%	3	10.0%

Participants classified as very low food security (VLFS) were significantly older (mean = 40.7 years) than those reporting high food security (mean = 17.2 years) or low food security (19.8 years). Intake of dietary protein from MF was significantly lower in those with VLFS (mean = 6.7 g/protein/day) compared to those with HFS (mean = 38.9 g/protein/day) and LFS (47.9 g/protein/day). Intake of intact dietary protein was significantly higher in those with VLFS (mean = 46.9 g/protein/day) compared to those with HFS (mean = 18.3 g/protein/day) and LFS (14.2 g/protein/day). In line with these findings, those with VLFS reported a significantly higher phenylalanine prescription (mean = 1670 mg/phenylalanine/day) and consumed more dietary phenylalanine (mean = 2037 mg/phenylalanine/day) than those with LFS, but not HFS or MFS. There was no significant difference in metabolic control between groups; however, those with MFS had the highest mean blood phenylalanine concentrations (1030 μmol/L) vs those with VLFS who had the lowest mean concentrations (319 μmol/L). There was no significant difference in medication use by group. Differences in variables by food security category, adjusted for medications and age, are reported in Table 3.

**Table 3:** Differences in participant characteristics by food security status

	High food security (HFS) (n = 15)	Marginal food security (MFS) (n = 3)	Low food security (LFS) (n = 9)	Very low food security (VLFS) (n = 3)	
	Mean (SD)	Mean (SD)	Mean (SD)	Mean (SD)	* *P*‐value
Age (years)	17.2 (4.6)^a^	28.3 (14.0)	19.8 (10.3)^a^	40.7 (22.7)^b^	.10
BMI (kg/m^2^)	26.5 (10.4)	28.5 (8.8)	26.4 (5.8)	30.1 (12.0)	.95
Blood phenylalanine (μmol/L)	502 (387)	1030 (552)	719 (329)	319 (307)	.25
Blood tyrosine (μmol/L)	44.9 (11.5)	44.8 (8.2)	41.8 (9.6)	33.7 (5.1)	.62
Blood phe:tyr ratio	11.7 (8.8)	22.1 (10.1)	18.7 (9.9)	10.3 (11.0)	.34
Dietary phenylalanine prescription (mg/day)	741 (630)	600 (304)	620 (524)^a^	1670 (1268)^b^	.60
Compliance with phenylalanine prescription (%)	129 (53)	160 (139)	107 (62)	192 (145)	.18
Medication use (% taking medication)**	53.3%	33.3%	44.4%	66.7%	.85
Dietary intake					
Calories (kcals/day)	1641 (399)	1502 (567)	1327 (207)	1796 (799)	.21
Phenylalanine (mg/day)	866 (730)	691 (284)	626 (518)^a^	2037 (1417)^b^	.14
Total protein (g/day)	57.1 (8.4)	46.6 (33.4)	62.1 (17.7)	53.6 (28.8)	.16
Intact protein (g/day)	18.3 (14.6)^a^	16.4 (3.0)	14.2 (11.2)^a^	46.9 (35.7)^b^	.07
MF protein (g/day)	38.9 (14.6)^b^	30.2 (36.3)	47.9 (20.2)^b^	6.7 (11.5)^a^	.04

*Note:* a is significantly less than b (Bonferroni *P*‐value <.05).

* Adjusted for age and medication use.

** Adjusted for age.

When comparing participants by food security status defined as food secure or food insecure, there were no significant differences in adjusted analyses. Mean calorie intake (FS = 1618 kcal/day vs FI = 1444 kcal/day; *P*‐value = .135) approached significance and warrants further study. Mean blood phenylalanine was also lower in food secure participants compared to food insecure participants (590 μmol/L vs 619 μmol/L), and mean blood tyrosine was higher (45 μmol/L vs 40 μmol/L), but not significantly so.

## 4 DISCUSSION

These pilot data suggest females with PKU have a higher prevalence of food insecurity compared to the general U.S. population (40% vs 11.1%). Adolescents with PKU had a higher prevalence of food insecurity (44%) compared to adults with PKU (36%). The prevalence of females with PKU experiencing very low food security (10%) is alarming, more than twice the prevalence in the United States in 2018. Adults residing in households with children also had a higher prevalence of food insecurity compared to the general U.S. population (66% vs 13.9%).

Participants with VLFS were older and consumed more dietary phenylalanine and intact protein and less MF protein compared to those with higher food security, even when adjusting for use of blood phenylalanine‐lowering medications. Blood phenylalanine concentrations were also lower in this group though this finding was not significant. Severity of PKU was not assessed as DNA samples were not collected, so the participants in the VLFS group may have had less severe mutations in the PAH gene. Interestingly, those with LFS (also classified as food insecure), were much more similar in all variables to those considered food secure compared with those considered VLFS. Future research should evaluate the experiences of food insecurity in this group in particular.

Due to the pilot nature of this study, many health outcomes associated with food insecurity in adolescent and adult populations were not examined. BMI was assessed but there was no difference by food security status. Food insecurity in children and adults can impact long‐term physical and mental health including depression and anxiety and indicators of diabetes, cardiovascular disease, and hypertension. Specific to PKU, those struggling with resources may choose not to spend money on critical components of PKU treatment including medical food, LPMF, and co‐pays for clinical care. In addition, the PKU diet stresses foods low in protein including fruits and vegetables, which may be considered too expensive by families experiencing financial strain. Such families may choose less expensive processed foods lower in protein but higher in carbohydrates and added sugars. This is also true in the general population where food insecurity is significantly associated with poor diet quality including higher consumption of sugar‐sweetened beverages and lower consumption of vegetables.^18^ Noncompliance with the PKU diet and poor diet quality related to food insecurity could exacerbate mental and physical health issues in an already vulnerable population.

There were several limitations to this study. Participants volunteered to attend the camp from which they were recruited for this study, potentially introducing self‐selection bias. Due to the small sample size, results may not be generalizable to the general PKU population and significant differences between groups may not have been detected, thus comparisons with the general U.S. population are descriptive only. Dietary intake was assessed by three‐day food records that can be inaccurate; however, all food records were reviewed with participants by a metabolic dietitian. Food insecurity was also measured over different periods in adolescents (30‐day reference period) and adults (12‐month reference period), following USDA recommendations. The USDA Household Food Security Survey Module does not define the relationship between adults and children in the household. These adults simply report the presence of an individual under 18 years of age in their household whether or not they are financially responsible. In addition, there were three 11‐year old participants in this study who completed the Self‐Administered Survey Module for Youth Ages 12 and older though the module is not yet validated for 11‐year old adolescents. In the future, larger studies should also assess household income and percent of income spent on food, use and cost of LPMF per month, and medical food expense and source of financial coverage for medical food and LPMF.

These pilot data suggest differences in dietary intake and metabolic control between food secure and food insecure females with PKU, but larger studies would be valuable, including enrolling males and more households with children. An international study of food insecurity in individuals with IMD with expensive medical and dietary treatment is also needed, since many countries differ in expense and financial coverage for products. Examining impacts of food insecurity on physical and mental health, in addition to metabolic control, in patients with PKU is critical. In addition, qualitatively examining PKU patients' experiences with food insecurity could provide valuable insight in prevention and resolution.

Based on these pilot results, dietitians and other metabolic clinicians may consider screening for food insecurity in patients with PKU and other IMD. The USDA has published short tools to assess food security including a six‐item short form^14^ and a two‐item screener.^19^ The two‐item screener is validated and indicates an individual or household is struggling with food insecurity if they answer yes to either of the following questions: “Within the past 12 months, we worried whether our food would run out before we got money to buy more” or “Within the past 12 months, the food we bought just did not last and we didn't have money to get more”.^19^ This screener would take less than 1 minute and is a feasible, validated method to assess food insecurity in all patients with disorders associated with a significant financial burden. Patients that screen at‐risk for food insecurity can receive appropriate resources including information about local food banks and pantries and food assistance programs including the Supplemental Nutrition Assistance Program (SNAP), Women, Infants, and Children (WIC), the National School Lunch Program (NSLP), and the Summer Food Service Program, among others.

Prevalence of food insecurity was higher in this sample of females with PKU compared to the general U.S. population. Given the strong links between food insecurity and adverse health outcomes, efforts to prevent and address food insecurity in families with individuals with PKU should be explored.

## CONFLICT OF INTEREST

Kathryn Coakley, Suzanne Porter‐Bolton, Mary Lauren Salvatore, Rosalynn Blair, and Rani Singh have no conflict of interest.

## AUTHOR CONTRIBUTIONS

K.E.C. and R.H.S. designed and planned research; K.E.C., M.L.S., S.P.B. conducted research; K.E.C. analyzed data; R.B.B. and R.H.S. recruited patients and directed the camp; R.H.S. and M.L.S. obtained I.R.B. approval; K.E.C. developed the manuscript; K.E.C. and R.H.S. had primary responsibility for final content. All authors read and approved the final manuscript.

## ETHICS APPROVAL

Approval for this study was granted by the Emory University Institutional Review Board (IRB).

## INFORMED CONSENT

All procedures followed were in accordance with the ethical standards of the responsible committee on human experimentation (institutional and national) and with the Helsinki Declaration of 1975, as revised in 2000 (5). Informed consent was obtained from all patients for being included in the study. Identifying information was not collected.

## ANIMAL RIGHTS

This article does not contain any studies with animal subjects performed by the any of the authors.

## AVAILABILITY OF DATA

The data that support the findings of this study are available from the corresponding author upon reasonable request.

## Appendix 1

*USDA Household Food Security Survey Module*

The following questions are about the food situation in your home *during the last 12 months*. Please circle the answer that best describes you.

1. I/we worried whether my/our food would run out before I/we got money to buy more.

[ ] Often true [ ] Sometimes true [ ] Never true [ ] Don't know

2. The food that I/we bought just didn't last, and I/we didn't have money to get more.

[ ] Often true [ ] Sometimes true [ ] Never true [ ] Don't know

3. I/we couldn't afford to eat balanced meals.

[ ] Often true [ ] Sometimes true [ ] Never true [ ] Don't know

4. In the last year, since June of last year, did you or other adults in your household ever cut the size of your meals or skip meals because there wasn't enough money for food?

[ ] Yes[ ] No [ ] Don't know

5. If you answered yes to question 4, how often did this happen?

[ ] Almost every month[ ] Some months but not every month [ ] Only 1 or 2 months [ ] Don't know

6. In the last 12 months, did you ever eat less than you felt you should because there wasn't enough money for food?

[ ] Yes[ ] No [ ] Don't know

7. In the last 12 months, were you every hungry but didn't eat because there wasn't enough money for food?

[ ] Yes[ ] No [ ] Don't know

8. In the last 12 months, did you lose weight because there wasn't enough money for food?

[ ] Yes[ ] No [ ] Don't know

9. In the last 12 months, did you or other adults in your household ever not eat for a whole day because there wasn't enough money for food?

[ ] Yes[ ] No [ ] Don't know

10. If you answered yes to question 9, how often did this happen?

[ ] Almost every month[ ] Some months but not every month [ ] Only 1 or 2 months[ ] Don't know

*End of Survey for households with NO children under age 18*.

*Continue for Households with CHILDREN UNDER 18 ONLY:*

The following questions are about the food situation for your child/children living in the household under age 18 *during the last 12 months*. Please circle the answer that best describes you.

11. I/we relied on only a few kinds of low‐cost food to feed my/our child/the children because I was/we were running out of money to buy food.

[ ] Often true [ ] Sometimes true [ ] Never true [ ] Don't know

12. I/we couldn't feed my/our child/the children a balanced meal, because I/we couldn't afford that.

[ ] Often true [ ] Sometimes true [ ] Never true [ ] Don't know

13. My/our child or children were not eating enough because I/we just couldn't afford enough food.

[ ] Often true [ ] Sometimes true [ ] Never true [ ] Don't know

14. In the last 12 months, since June of last year, did you ever cut the size of your child's/any of the children's meals because there wasn't enough money for food?

[ ] Yes[ ] No [ ] Don't know

15. In the last 12 months, did any of the children ever skip meals because there wasn't enough money for food?

[ ] Yes[ ] No [ ] Don't know

16. If you answered yes to question 15, how often did this happen?

[ ] Almost every month

[ ] Some months but not every month

[ ] Only 1 or 2 months

[ ] Don't know

17. In the last 12 months, were your child/any of the children ever hungry but you just couldn't afford more food?

[ ] Yes[ ] No [ ] Don't know

18. In the last 12 months, did your child/any of the children ever not eat for a whole day because there wasn't enough money for food?

[ ] Yes[ ] No [ ] Don't know

*USDA Self‐Administered Food Security Survey Module for Children Ages 12 Years and Older*

The following questions are about the food situation in your home *during the last month*. Please circle the answer that best describes you. Your answers will remain a secret.

1. Did you worry that food at home would run out before your family got money to buy more?

_______ A LOT _______ SOMETIMES _______ NEVER

2. Did the food that your family bought run out, and you didn't have money to get more?

_______ A LOT _______ SOMETIMES _______ NEVER

3. Did your meals only include a few kinds of cheap foods because your family was running out of money to buy food?

_______ A LOT _______ SOMETIMES _______ NEVER

4. How often were you not able to eat a balanced meal because your family didn't have enough money?

_______ A LOT _______ SOMETIMES _______ NEVER

5. Did you have to eat less because your family didn't have enough money to buy food?

_______ A LOT _______ SOMETIMES _______ NEVER

6. Has the size of your meals been cut because your family didn't have enough money for food?

_______ A LOT _______ SOMETIMES _______ NEVER

7. Did you have to skip a meal because your family didn't have enough money for food?

_______ A LOT _______ SOMETIMES _______ NEVER

8. Were you hungry but didn't eat because your family didn't have enough food?

_______ A LOT _______ SOMETIMES _______ NEVER

9. Did you not eat for a whole day because your family didn't have enough money for food?

_______ A LOT _______ SOMETIMES _______ NEVER
